# Proton Nuclear Magnetic Resonance Metabolomics Corroborates Serine Hydroxymethyltransferase as the Primary Target of 2-Aminoacrylate in a *ridA* Mutant of Salmonella enterica

**DOI:** 10.1128/mSystems.00843-19

**Published:** 2020-03-10

**Authors:** Andrew J. Borchert, Goncalo J. Gouveia, Arthur S. Edison, Diana M. Downs

**Affiliations:** a Department of Microbiology, University of Georgia, Athens, Georgia, USA; b Department of Biochemistry, University of Georgia, Athens, Georgia, USA; c Department of Genetics, Institute of Bioinformatics, and Complex Carbohydrate Center, University of Georgia, Athens, Georgia, USA; University of Sao Paulo

**Keywords:** ^1^H NMR, 2-aminoacrylate stress, RidA, metabolomics

## Abstract

The reactive intermediate deaminase RidA (EC 3.5.99.10) is conserved across all domains of life and deaminates reactive enamine species. When Salmonella enterica *ridA* mutants are grown in minimal medium, 2-aminoacrylate (2AA) accumulates, damages several pyridoxal 5′-phosphate (PLP)-dependent enzymes, and elicits an observable growth defect. Genetic studies suggested that damage to serine hydroxymethyltransferase (GlyA), and the resultant depletion of 5,10-methelenetetrahydrofolate (5,10-mTHF), was responsible for the observed growth defect. However, the downstream metabolic consequence from GlyA damage by 2AA remains relatively unexplored. This study sought to use untargeted proton nuclear magnetic resonance (^1^H NMR) metabolomics to determine whether the metabolic state of an S. enterica *ridA* mutant was accurately reflected by characterizing growth phenotypes. The data supported the conclusion that metabolic changes in a *ridA* mutant were due to the IlvA-dependent generation of 2AA, and that the majority of these changes were a consequence of damage to GlyA. While many of the metabolic differences for a *ridA* mutant could be explained, changes in some metabolites were not easily modeled, suggesting that additional levels of metabolic complexity remain to be unraveled.

**IMPORTANCE** The accumulation of the reactive enamine intermediate 2-aminoacrylate (2AA) elicits global metabolic stress in many prokaryotes and eukaryotes by simultaneously damaging multiple pyridoxal 5′-phosphate (PLP)-dependent enzymes. This work employed ^1^H NMR to expand our understanding of the consequence(s) of 2AA stress on metabolite pools and effectively identify the metabolic changes stemming from one damaged target: GlyA. This study shows that nutrient supplementation during ^1^H NMR metabolomics experiments can disentangle complex metabolic outcomes stemming from a general metabolic stress. Metabolomics shows great potential to complement classical reductionist approaches to cost-effectively accelerate the rate of progress in expanding our global understanding of metabolic network structure and physiology. To that end, this work demonstrates the utility in implementing nutrient supplementation and genetic perturbation into metabolomics workflows as a means to connect metabolic outputs to physiological phenomena and establish causal relationships.

## Abstract (precis)

The accumulation of the reactive enamine intermediate 2-aminoacrylate (2AA) elicits global metabolic stress in many prokaryotes and eukaryotes by simultaneously damaging multiple pyridoxal 5′-phosphate (PLP)-dependent enzymes. This work employed ^1^H NMR to expand our understanding of the consequence(s) of 2AA stress on metabolite pools and effectively identify the metabolic changes stemming from one damaged target: GlyA. This study shows that nutrient supplementation during ^1^H NMR metabolomics experiments can disentangle complex metabolic outcomes stemming from a general metabolic stress. Metabolomics shows great potential to complement classical reductionist approaches to cost-effectively accelerate the rate of progress in expanding our global understanding of metabolic network structure and physiology. To that end, this work demonstrates the utility in implementing nutrient supplementation and genetic perturbation into metabolomics workflows as a means to connect metabolic outputs to physiological phenomena and establish causal relationships.

## INTRODUCTION

The metabolic state of the cell at a given time reflects the cumulative result of inputs to the system of cellular metabolism that include, but are not limited to, transcription, translation, enzyme activity, and metabolic flux. Deconvoluting the role of a specific cellular process in this complex network requires both global and local knowledge, acquired by the integration of multidisciplinary approaches (1, 2). Metabolomics approaches have been successful in accelerating the elucidation of complex metabolic and physiological states of an organism and help complement biochemical and genetic approaches that can require significant resources and time (1, 3–7). Metabolomics has the benefit of providing a snapshot of all metabolic changes in a system without requiring that these differences produce an observable (growth) phenotype. The integration of large metabolomics data sets with reductionist biochemical genetic analyses is advantageous, since the former allows the detection of underlying metabolic changes caused by genetic or environmental perturbation, while data from the latter provides biological relevancy to frame conclusions. The RidA paradigm of endogenous metabolic stress provides an opportunity to explore the utility of integrating metabolomics analysis with the biochemical genetic approaches that have defined the framework of this stress.

**FIG 1:**
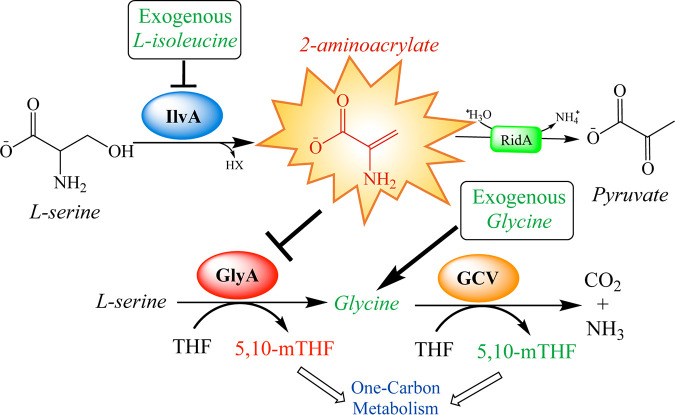
RidA paradigm of 2-aminoacrylate stress in S. enterica. Biosynthetic serine/threonine dehydratase (IlvA) catalyzes the β-elimination of l-serine to generate the reactive enamine 2-aminoacrylate (2AA). The activity of IlvA is prevented via allosteric inhibition by l-isoleucine. 2AA is hydrolyzed to pyruvate by the reactive intermediate deaminase A (RidA). In the absence of RidA, 2AA accumulates and can damage a number of PLP-dependent enzymes. The most physiologically sensitive target of 2AA damage in S. enterica grown in minimal glucose medium is serine hydroxymethyltransferase (GlyA), as judged by nutrient supplementation (9, 12). GlyA is responsible for the reversible transfer of the hydroxymethyl from serine to tetrahydrofolate (THF), generating glycine and 5,10-methylenetetrahydrofolate (5,10-mTHF). The glycine cleavage complex (GCV) can further catabolize glycine, generating additional 5,10-mTHF.

Pyridoxal 5′-phosphate (PLP)-dependent α,β-eliminases generate reactive enamine species as important reaction intermediates from amino acid substrates. A subset of these α,β-eliminases release reactive enamine intermediates into the cellular milieu. RidA (reactive intermediate deaminase A) catalyzes the deamination of free enamine species. 2-Aminoacrylate (2AA) is a reactive enamine species generated from l-serine by the biosynthetic serine/threonine dehydratase (IlvA; EC 4.3.1.19) (8). The absence of RidA from Salmonella enterica allows the 2AA produced by IlvA to accumulate and damage multiple other PLP-dependent enzymes, generating a number of detectable mutant phenotypes (8–11). Relevant to this study, serine hydroxymethyltransferase (GlyA; EC 2.1.2.1) is the most physiologically significant target for 2AA damage in S. enterica, since its damage causes a growth-limiting reduction in 5,10-methylenetetrahydrofolate (5,10-mTHF) (12). Importantly, exogenous glycine can bypass the 5,10-mTHF limitation and restore growth by allowing 5,10-mTHF production via the glycine cleavage complex (GCV). IlvA is subject to allosteric control by l-isoleucine; thus, exogenous l-isoleucine restores growth to a *ridA* mutant by preventing 2AA generation (13–15). Therefore, isoleucine and glycine supplements provide mechanistically distinct means to restore full growth to an S. enterica *ridA* mutant. With the former, the 2AA stress is eliminated, and with the latter, one impact from the stress is circumvented. A summary of the RidA paradigm for S. enterica is provided in Fig. 1.

Damage to GlyA by 2AA perturbs glycine and 5,10-mTHF synthesis, but the extent of the changes to the global metabolic network caused by this perturbation is less clear. In this study, untargeted proton nuclear magnetic resonance (^1^H NMR) metabolomics and nutrient supplementation were used to dissect the global metabolic consequences associated with the accumulation of 2AA, extending those deduced from past growth studies. ^1^H NMR was used to measure the endogenous and exogenous (e.g., spent culture medium) metabolomes of S. enterica wild-type and *ridA* mutant strains grown in various media. The strengths of NMR-based metabolomics, including simple sample preparation, broad chemical coverage, confident chemical assignments, and straightforward quantification of metabolites, benefited this study (7, 16–18). The data showed a clear metabolic fingerprint associated with an S. enterica *ridA* mutant grown in minimal glucose medium. Significantly, the addition of isoleucine to the growth medium restored the fingerprint to that of the wild-type strain. Further, the addition of glycine to the growth medium almost completely rescued the *ridA* fingerprint back to that of the wild type, suggesting that the primary impact of 2AA stress is via damaged GlyA. Importantly, this conclusion could not be reached from biochemical genetic data alone. Overall, this work demonstrates the potential for appropriate metabolomics experiments, in combination with biochemical genetic insights, to dissect perturbations to the metabolic network and isolate systems and subsystems impacted by these perturbations.

## RESULTS AND DISCUSSION

### Metabolic differences in a *ridA* mutant are a consequence of IlvA-dependent 2AA generation.

**FIG 2:**
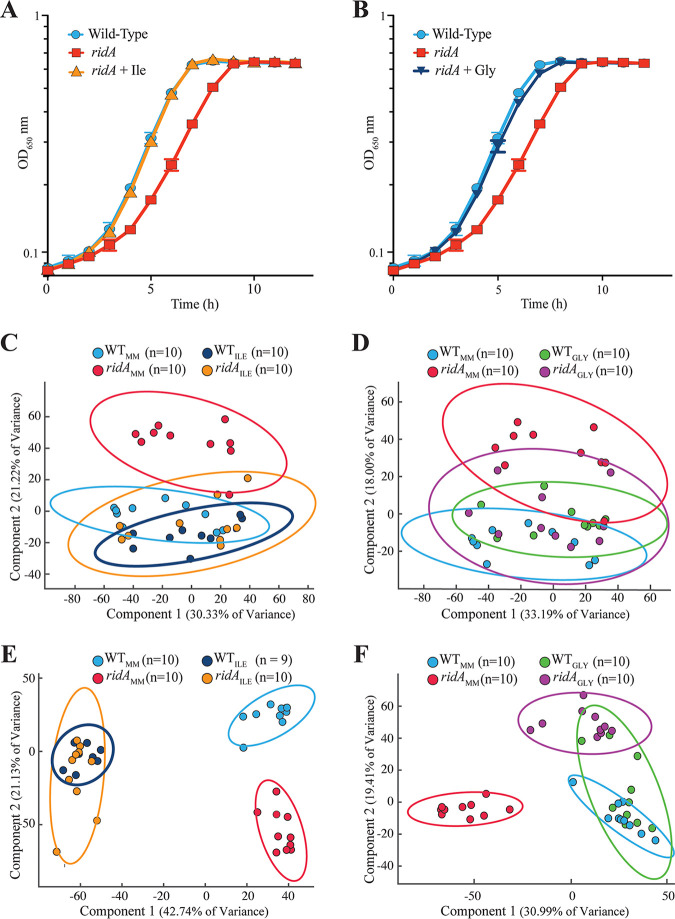
Isoleucine and glycine restore growth and metabolic stability to a *ridA* mutant. S. enterica wild-type and *ridA* mutant strains were grown at 37°C in minimal glucose (11 mM) medium. Supplementation of the medium with 1 mM l-isoleucine (A) or 1 mM glycine (B) restored wild-type growth to a *ridA* mutant. Data are means from three biological replicates, where error bars represent the 95% confidence intervals. OD_650_, optical density at 650 nm. Principle component analysis (PCA) score plots show separation of endogenous (C and D) and exogenous (E and F) metabolite profiles for S. enterica wild-type and *ridA* mutant strains following 16 h of growth in minimal glucose medium at 37°C. Metabolomes obtained from growth with supplementation with isoleucine (C and E) or glycine (D and F) are shown. Colored ellipses represent the 95% confidence intervals for each group.

In an S. enterica *ridA* mutant, 2AA accumulates and damages a number of PLP-dependent enzymes, causing a mild growth defect in minimal glucose medium (Fig. 2A and B). Both the metabolome and transcriptome of a *ridA* mutant differ from a wild-type strain grown in minimal medium (19, 20). The growth phenotypes associated with an S. enterica *ridA* mutant result from the accumulation of 2AA, and all RidA orthologs described to date share enamine/imine deaminase activity (8, 11, 21, 22). Other functions for RidA have been proposed, including a role as a translational inhibitor/RNase (23–26) or a molecular chaperone (27–29). Because of the potential for multiple RidA-associated functions, it was important to determine whether the metabolic restructuring of a *ridA* mutant was caused by 2AA accumulation. The endogenous and exogenous metabolomes of a *ridA* mutant (DM3480) were compared to those obtained for the isogenic parental strain (DM9404, wild type) under multiple growth conditions using untargeted ^1^H NMR metabolomics. Principle component analysis (PCA) showed the separation of the *ridA* mutant and wild-type-associated metabolomes (both exogenous and endogenous) following growth on minimal glucose medium (Fig. 2C to F).

In an S. enterica *ridA* mutant grown in minimal glucose medium, IlvA acts as the dominant, if not sole, generator of 2AA (8). l-Isoleucine allosterically inhibits IlvA, lowering activity, reducing the production of 2AA, and restoring wild-type growth to the *ridA* mutant (8) (Fig. 2A). Therefore, the presence of l-isoleucine in the medium will eliminate any metabolic effects that are the consequence of 2AA accumulation. The endogenous and exogenous metabolomes of *ridA* and wild-type strains grown in the presence of 1 mM exogenous l-isoleucine were not distinguishable based on PCA analyses (Fig. 2C and E). These data supported the conclusion that differences found in the metabolomes of the strains in minimal medium were the result of 2AA accumulation. This result was the first to demonstrate that the elimination of detectable *ridA* mutant phenotypes was mirrored by the restoration of metabolite balance. Significantly, this conclusion reinforced that a valid interpretation of the system had been obtained by the biochemical genetic analyses reported previously.

### Bypassing the one-carbon starvation in a *ridA* mutant eliminates most, but not all, metabolic changes.

When 2AA accumulates in an S. enterica *ridA* mutant, it damages multiple PLP-dependent enzymes by covalently modifying PLP in the active site. One of the target enzymes is GlyA, whose activity is reduced to ∼20% of wild-type activity in minimal glucose medium (9, 12). GlyA catalyzes the transfer of the hydroxymethyl group from l-serine substrate to THF, forming glycine and 5,10-mTHF (Fig. 1). Damage of GlyA by 2AA causes the growth defect of an S. enterica *ridA* mutant in minimal medium, leading to its designation as the most physiologically sensitive target in this organism (9, 12). The addition of glycine to the medium restores growth of a *ridA* mutant, since 5,10-mTHF can be generated from glycine by the glycine cleavage system (GCV) and bypasses the need for GlyA (12) (Fig. 1 and 2B). Growth of a *ridA* mutant in the presence of glycine was expected to restore a subset of the *ridA* metabolome back to that of the wild type. Further, the metabolites in this subset would define the metabolic subsystem that was perturbed by the reduction or lack of GlyA-dependent formation of glycine/5,10-mTHF. With this logic, the metabolomes of strains grown with glycine were used to distinguish between the metabolic effects resulting from 2AA-dependent damage of GlyA and those resulting from other 2AA-dependent perturbations. PCA showed that, when the cells were grown in the presence of 1 mM glycine, the endogenous metabolome of the *ridA* mutant strain was no longer distinguishable from that of the wild type (Fig. 2D). Surprisingly, these data indicated that the majority of the metabolic perturbations, at least those detected by ^1^H NMR, in the endogenous metabolome of a *ridA* mutant strain were downstream effects of the damage to GlyA. Consistent with this finding, PCA of the external metabolome of a *ridA* mutant grown with glycine and the corresponding metabolome from the wild type showed an obvious decrease in the separation of metabolic signatures, although the separation was not eliminated, as it was with isoleucine.

In total, the data shown in Fig. 2 supported the conclusions that (i) ^1^H NMR metabolomics detected a molecular signature unique to an S. enterica *ridA* mutant strain, (ii) the deviation in molecular signatures between *ridA* and wild-type strains was dependent upon the generation of 2AA by IlvA, and (iii) the metabolic consequences of 2AA-dependent GlyA damage dominated the detectable differences between the *ridA* and wild-type strains. Since multiple other targets exist in S. enterica for damage by 2AA, the latter conclusion suggests that (i) damage of the other targets (i.e., IlvE) have a limited impact on the overall metabolic profile or (ii) the spectral resolution and/or sensitivity of these methods failed to uncover all the metabolic perturbations stemming from 2AA stress.

### 2AA stress influences amino acid metabolism and mixed-acid fermentation.

**FIG 3:**
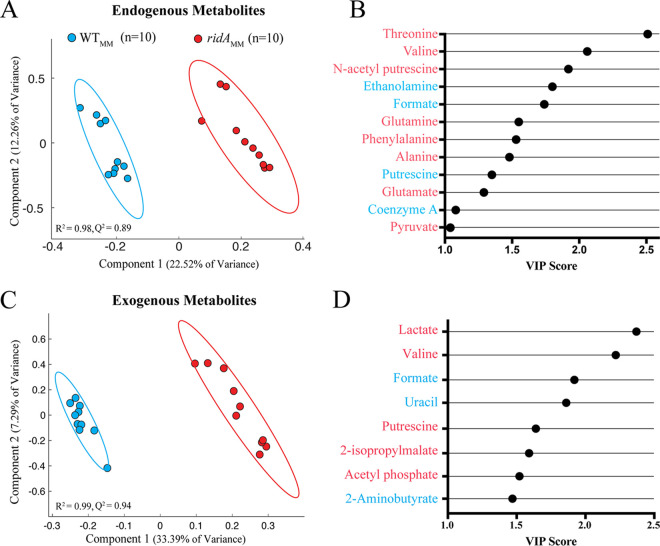
Partial least-squares discriminant analysis (PLS-DA) highlights separation of metabolomic profiles. PLS-DA score plots show clear separation by PLS component 1 of wild-type (*n* = 10, blue) and *ridA* mutant (*n* = 10, red) endogenous (A) and exogenous (B) metabolite samples following growth in minimal glucose medium. Variable importance of projection (VIP) scores were plotted for the metabolites that contributed significantly (VIP of >1) to separation by PLS-DA component 1 for endogenous (C) and exogenous (D) samples. VIP scores were determined as the average from all peak VIP scores belonging to the given metabolite. Metabolites colored blue were elevated in wild-type samples, while those colored red were elevated in *ridA* mutant samples.

**FIG 4:**
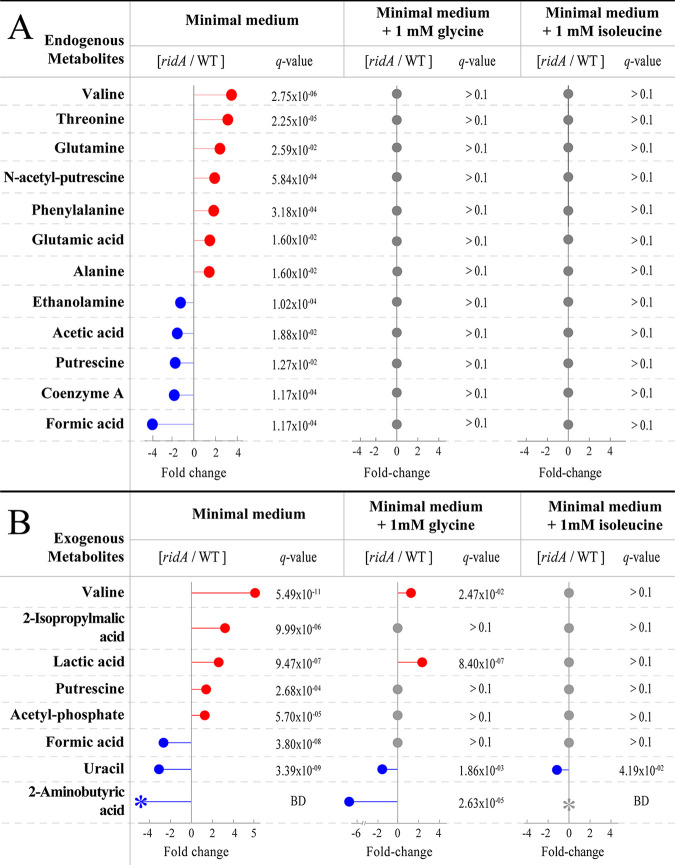
Significantly altered metabolites under different conditions. Fold change differences between *ridA* mutant and wild-type strains were calculated for both intracellular metabolites (A) and external metabolites (B). Red circles indicate higher abundance in *ridA* mutants, blue circles indicate high abundance in the wild type, and gray circles indicate no significant (*q* value of <0.1) difference in metabolite abundance between *ridA* mutants and wild-type samples. *q* values represent false discovery rate-corrected *P* values according to the Benjamini-Hochberg method (51). Colored asterisks, with corresponding “BD” *q* value designation, specify that a fold change was not determined, since the feature in *ridA* (blue) or both (gray) groups was below the detection (BD) limit for multiple samples.

In total, sixteen endogenous and ten exogenous metabolites that we could quantify by integration were identified from the NMR spectra, and their patterns were considered in the context of S. enterica physiology (see Tables S1 and S2 and Fig. S1 and S2 in the supplemental material). Partial least-squares discriminant analysis (PLS-DA) score plots were used to identify the specific metabolic pathways perturbed by 2AA stress and further understand the metabolic differences between *ridA* mutant and wild-type strains when grown in minimal medium (Fig. 3A and C). Variable importance in projection (VIP) scores were determined for all NMR features in the endogenous and exogenous data sets that contributed most to the PLS-DA separation in the first component (Tables S3 and S4) (30). From these data, VIP plots were created with the identified metabolites that contributed to the separation of endogenous (Fig. 3B) and exogenous (Fig. 3D) metabolomes. Importantly, the VIP scores reported in these plots represent the average VIP scores taken from all the quantifiable peaks comprising a given metabolite. VIP scores of >1 indicated that elevated threonine, valine, *N*-acetyl putrescine, glutamine, phenylalanine, alanine, glutamate, and pyruvate and diminished ethanolamine, formate, putrescine, and coenzyme A (CoA) drove PLS-DA separation of *ridA* endogenous metabolomes (Fig. 3B). Similarly, VIP analysis revealed that elevated lactate, valine, putrescine, 2-isopropylmalate, and acetyl-phosphate and diminished formate, uracil, and 2-aminobutyrate drove PLS-DA separation of *ridA* exogenous metabolomes (Fig. 3D). In total, these data indicated that metabolites in amino acid metabolism and mixed-acid fermentation were largely responsible for the separation of the samples, as determined by PLS-DA analysis. The integration of peaks corresponding to all identifiable metabolites and comparison by Student’s unpaired two-sample *t* test showed that 12 of 16 endogenous metabolites and 8 of 10 exogenous metabolites were significantly altered in a *ridA* mutant strain (*q* value of <0.1) (Fig. 4 and Tables S5 and S6). Overall, the VIP analysis agreed well with the findings from univariate analysis of the ^1^H NMR data set, as only endogenous pyruvate had a VIP score of >1 but did not differ significantly by univariate analysis (*q* value of >0.1), and only endogenous acetate differed significantly by univariate analysis but failed to meet a VIP score of >1.

10.1128/mSystems.00843-19.1 FIG S1 ^1^H NMR spectra for endogenous samples. (A) Endometabolome overlaid spectra of wild-type samples (*n* = 10, blue) and *ridA* mutant samples (*n* = 10, red) after growth in minimal medium. Expansions from 0 to 4.5 ppm (B) and 5 to 9.5 ppm (C) of the mean spectra obtained from wild-type and *ridA* endogenous samples following growth in minimal medium containing isoleucine and minimal medium containing glycine are also provided. The water region was removed and not displayed (4.7 to 4.8 ppm). Annotations: 1, CoA; 2, valine; 3, threonine; 4, alanine; 5, putrescine; 6, acetic acid; 7, glutamic acid; 8, ethanolamine; 9, *N*-acetyl-putrescine; 10, uracil; 11, phenylalanine; 12, formic acid. Download FIG S1, PDF file, 0.6 MB. Copyright © 2020 Borchert et al. 2020 Borchert et al. This content is distributed under the terms of the Creative Commons Attribution 4.0 International license.

10.1128/mSystems.00843-19.2 FIG S2 ^1^H NMR spectra for exogenous samples. (A) Exometabolome overlaid spectra of wild-type samples (*n* = 10, blue) and *ridA* mutant samples (*n* = 10, red), grown in minimal medium. Expansions from 0 to 4.5 ppm (B) and 5 to 9.5 ppm (C) of the mean spectra obtained from wild-type and *ridA* exogenous samples following growth in minimal medium containing isoleucine and minimal medium containing glycine are also provided. The residual water resonance between 5.1 and 5.7 ppm (due to proton exchange with large amounts of formic acid) was removed during processing. Annotations: 1, isopropylmalic acid; 2, valine; 3, lactic acid; 4, putrescine; 5, acetic acid; 6, acetyl phosphate; 7, uracil; 8, formic acid. Download FIG S2, PDF file, 0.4 MB. Copyright © 2020 Borchert et al. 2020 Borchert et al. This content is distributed under the terms of the Creative Commons Attribution 4.0 International license.

10.1128/mSystems.00843-19.4 TABLE S1 Endogenous metabolites identified by ^1^H-NMR in pellet samples with confidence levels. Download Table S1, PDF file, 0.1 MB. Copyright © 2020 Borchert et al. 2020 Borchert et al. This content is distributed under the terms of the Creative Commons Attribution 4.0 International license.

10.1128/mSystems.00843-19.5 TABLE S2 Endogenous metabolites identified by ^1^H-NMR in medium samples with confidence levels. Download Table S2, PDF file, 0.1 MB. Copyright © 2020 Borchert et al. 2020 Borchert et al. This content is distributed under the terms of the Creative Commons Attribution 4.0 International license.

10.1128/mSystems.00843-19.6 TABLE S3 VIP scores for endogenous PLS-DA plot component 1. Download Table S3, PDF file, 0.1 MB. Copyright © 2020 Borchert et al. 2020 Borchert et al. This content is distributed under the terms of the Creative Commons Attribution 4.0 International license.

10.1128/mSystems.00843-19.7 TABLE S4 VIP scores for exogenous PLS-DA plot component 1. Download Table S4, PDF file, 0.1 MB. Copyright © 2020 Borchert et al. 2020 Borchert et al. This content is distributed under the terms of the Creative Commons Attribution 4.0 International license.

10.1128/mSystems.00843-19.8 TABLE S5 Endogenous metabolites integration values and descriptive statistics. Download Table S5, PDF file, 0.1 MB. Copyright © 2020 Borchert et al. 2020 Borchert et al. This content is distributed under the terms of the Creative Commons Attribution 4.0 International license.

10.1128/mSystems.00843-19.9 TABLE S6 Exogenous metabolites integration values and descriptive statistics. Download Table S6, PDF file, 0.1 MB. Copyright © 2020 Borchert et al. 2020 Borchert et al. This content is distributed under the terms of the Creative Commons Attribution 4.0 International license.

The integration of the peaks described above from the samples grown in minimal glucose medium supplemented with isoleucine showed that concentrations of the altered metabolites were restored to wild-type levels (Fig. 4 and Table S5). The only exception was of the abundance of exogenous uracil, which was 3.1-fold lower in a *ridA* mutant than the wild type following growth in minimal glucose medium and only 1.3-fold lower when grown in minimal glucose medium containing isoleucine. This discrepancy might be a consequence of the fact that the doublet integrated to define uracil concentration was poorly resolved in three of the wild-type samples from growth with isoleucine, possibly causing an overestimation of the mean peak area for the wild type (Fig. S3). Nonetheless, isoleucine clearly reversed the metabolic differences observed for a *ridA* mutant grown in minimal glucose medium. These results further supported the conclusion from the PCA plots that the metabolic perturbations detected with ^1^H NMR were the consequence of IlvA-dependent 2AA stress.

10.1128/mSystems.00843-19.3 FIG S3 Spectral distortions for samples grown with isoleucine at the uracil region. (A) Exometabolome overlaid spectra expansion from 5.76 ppm to 7.7 ppm of wild-type (*n* = 10, blue) and *ridA* mutant (*n* = 10, orange) samples following growth in minimal medium containing isoleucine. (B) Expansion from 7.58 to 7.66 ppm illustrates the uracil peak used for integration. Distortions of the doublet peak shape are noted for a portion of the wild-type samples. In addition, alignment artifacts create peak shape changes for a portion of the *ridA* samples. These two factors contribute to the area under the curve calculation. Download FIG S3, PDF file, 0.2 MB. Copyright © 2020 Borchert et al. 2020 Borchert et al. This content is distributed under the terms of the Creative Commons Attribution 4.0 International license.

### CoA limitation in a *ridA* mutant is captured by untargeted ^1^H NMR metabolomics.

During growth on minimal glucose medium, S. enterica derives most of its one-carbon units from serine via the generation of 5,10-mTHF by the PLP-dependent enzyme, GlyA (31). GlyA activity is decreased more than 5-fold in a *ridA* mutant compared to that of the wild type, as a result of damage by 2AA (9). The constraint on GlyA activity leads to significantly decreased CoA (3-fold) in a *ridA* mutant, since the biosynthesis of CoA involves the 5,10-mTHF-dependent enzyme 3-methyl-2-oxobutanoate hydroxymethyltransferase (PanB; EC 2.1.2.11) (9). Gratifyingly, the untargeted metabolomic experiments here captured the lowered CoA levels in a *ridA* mutant (Fig. 4). Furthermore, the addition of glycine to the growth medium eliminated the difference in CoA levels between the *ridA* mutant and the wild type (Fig. 4).

### 2AA-dependent decrease in 5,10-mTHF generates additional metabolic effects.

Glycine supplementation eliminated most of the metabolic changes observed for a *ridA* mutant, supporting the conclusion that these changes were a consequence of the constrained function of GlyA. The known endogenous metabolites restored to wild-type levels included valine, threonine, glutamine, *N*-acetyl putrescine, phenylalanine, glutamic acid, alanine, ethanolamine, acetic acid, CoA, putrescine, and formic acid (Fig. 4A), as well as exogenous 2-isopropylmalic acid, putrescine, acetyl-phosphate, and formic acid (Fig. 4B). While the majority of metabolites were restored to balance, a few exogenous metabolites were not, notably valine and uracil. The difference between *ridA* mutant and wild-type valine content was partially reduced (*q* value < 0.1) when glycine was included in the growth medium (5.0-fold higher to 1.4-fold higher in the *ridA* background); similarly, the difference between *ridA* mutant and wild-type uracil content was also reduced (*q* value < 0.1) when glycine was present in the growth medium (3.1-fold lower to 1.5-fold lower in the *ridA* background). These findings suggested that the state of 5,10-mTHF was partially responsible for the concentration change in valine and uracil during 2AA stress (Fig. 4 and Table S6).

**FIG 5:**
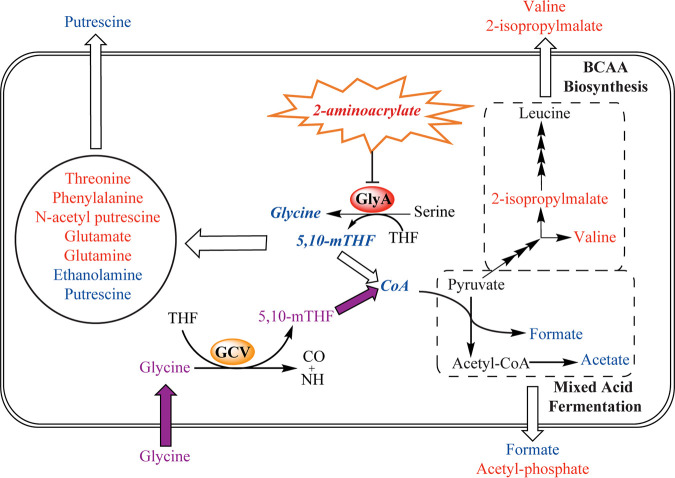
Working model for metabolic outcomes resulting from 5,10-mTHF limitation. 2AA-dependent damage of GlyA in a *ridA* mutant of S. enterica causes a glycine/5,10-mTHF limitation. The 5,10-mTHF limitation leads to a downstream decrease in CoA, altering flux through CoA-dependent mixed acid fermentation. In this working model, accumulated pyruvate is rerouted toward branched-chain amino acid (BCAA) biosynthesis. The metabolites on the left side of the cell schematic are altered by an unknown, but glycine/5,10-mTHF-dependent, mechanism. Metabolites colored blue are elevated in the wild type, and those colored red are elevated in the *ridA* mutant. The purple pathway represents the effect of supplemented glycine in restoring endogenous glycine/5,10-mTHF levels. Metabolites that are boldfaced and italicized were not detected by ^1^H NMR. Compounds listed outside the rounded rectangle represent metabolites detected by ^1^H NMR in the growth medium.

A *ridA* mutant accumulated less endogenous formic acid and acetic acid than the wild-type strain (Fig. 4A). The finding that these trends were eliminated by the presence of glycine in the growth medium suggested a model where CoA limitation triggered a change in flux through mixed-acid fermentation (Fig. 5). During mixed-acid fermentation, pyruvate-formate lyase (PflB; EC 2.3.1.54) uses CoA and pyruvate as substrates for the production of formate and acetyl-CoA, which is further processed to acetate (32). A bottleneck in CoA biosynthesis would reduce PflB-dependent formation of formate and downstream production of acetate. The accumulation of pyruvate during late-exponential-phase growth in a *ridA* mutant is consistent with this model (9). An increase in endogenous pyruvate in a *ridA* mutant was suggested by the PLS-DA model; however, the increase did not meet the threshold for statistical significance (*q* value of 0.12) and, thus, was not discussed (Table S5). Two molecules of pyruvate are used during valine synthesis, and in Klebsiella aerogenes pyruvate accumulation increases valine production (33, 34). The increase in endogenous and exogenous valine and exogenous 2-isopropylmalic acid, which is formed from an intermediate in valine biosynthesis, in a *ridA* mutant is eliminated by growth in glycine (Fig. 4B). Therefore, the increase in valine and 2-isopropylmalic acid content indicates that overflow pyruvate in a *ridA* mutant is diverted toward valine synthesis (Fig. 4 and 5).

### Conclusions.

The untargeted ^1^H NMR metabolomics approach used here exposed the global metabolic consequences of eliminating RidA from S. enterica. Both endogenous and exogenous metabolomes were assessed, and among the multitude of features visualized, 16 endogenous and 10 exogenous metabolites were confidently identified. Multivariate analysis by PCA and PLS-DA showed a clear difference between the metabolomes of *ridA* and wild-type strains grown in minimal medium. Importantly, the PLS-DA models revealed a fingerprint for a *ridA* metabolome, and the high goodness-of-fit (*Q*^2^) scores from cross-validation showed that these models effectively captured the metabolic separation between the two groups. A VIP score cutoff of >1 was used to identify metabolites contributing to separation of the respective fingerprints. The findings from VIP analysis also agreed well with the findings from univariate analysis of the ^1^H NMR data set. Altogether, these data indicated the two PLS-DA models, without the need for orthogonal signal correction (35), accounted for most identifiable and significantly altered metabolites and effectively separated wild-type metabolomes from those associated with *ridA* mutants.

The ^1^H NMR analyses of strains grown in medium containing isoleucine demonstrated that the bulk of metabolic changes between the *ridA* and wild-type strains are due to the IlvA-dependent generation of 2AA. This result significantly extended our understanding of the influence of 2AA on the metabolic network of a *ridA* mutant by showing the restoration of most metabolic feature discrepancies, including those that were not associated with a detectable growth phenotype. The growth defect of a *ridA* mutant is a consequence of damage to GlyA by 2AA (9, 12). The PCA of the ^1^H NMR data showed that the addition of glycine to the growth medium, which is expected to bypass the requirement for GlyA activity, largely restored the *ridA* mutant metabolome to wild-type levels. Univariate statistical analysis revealed the glycine-dependent restoration back to wild-type levels for 12/12 endogenous and 4/8 exogenous identifiable metabolites perturbed in the *ridA* mutant. These data supported a connection between GlyA damage by 2AA and changes in mixed-acid fermentation and branched-chain amino acid (BCAA) metabolism, as depicted in Fig. 5. The ^1^H NMR data found metabolic differences that were not easily modeled as a consequence of lowered 5,10-mTHF or CoA. Further dissection of the network generating these changes could incorporate the supplementation of growth medium with pantothenate, which restores CoA levels but not 5,10-mTHF levels (9).

Since fewer metabolites are present in spent medium samples, the spectra associated with the exogenous metabolome had less spectral overlap. Medium samples also did not require homogenization or methanol extraction, making their processing more expedient and straightforward. Therefore, the analysis of exogenous samples may offer a high-throughput and simplified way to continue characterization of the RidA paradigm. Such simplified and expedited analysis would be particularly useful during time course experiments or studies containing more genetic backgrounds and/or medium conditions, where dozens to hundreds of samples may be required.

Overall, the combination of ^1^H NMR metabolomics and relevant nutrient supplementation was successful in expanding the RidA/2-aminoacrylate paradigm in S. enterica and in making first steps toward delineating downstream consequences of GlyA damage from metabolic effects independent of glycine/5,10-mTHF perturbation. Historically, metabolomics approaches have been valuable in identifying correlations to generate hypotheses/models; however, the design of metabolomics experiments to act as a high-throughput means of testing these models and identifying mechanistic/causal relationships is a nascent field (36, 37). The RidA system provides an interesting case study in refining the experimental approach of metabolomics studies to include genetic manipulations and nutrient supplementations as a means to probe the underlying factors contributing to the metabolic differences observed following a perturbation. The study here highlighted (i) the benefit of using spent growth medium as an initial proxy for metabolome differences and (ii) the value of nutritional supplementation as a way to help define metabolic subnetworks. If applied to the study of complex metabolic systems, these approaches have the potential to contribute to understanding how a network responds to perturbation and drive our understanding of gene function and the physiological impact of various cellular components.

## MATERIALS AND METHODS

### Bacterial strains, chemicals, and media.

Strains used in this work are derivatives of Salmonella enterica subsp. *enterica* serovar Typhimurium strain LT2. The construction of the *ridA* null mutant (DM3480; *ridA3*::MudJ1734) is described elsewhere (38, 39). The wild-type strain used in this work (DM9404) is an LT2 derivative isogenic to the *ridA* mutant. Rich medium was Difco nutrient broth (NB) (8 g/liter) supplemented with 5 g/liter NaCl. No-carbon E medium (NCE) containing 1 mM MgSO_4_ (40), trace metals (41), and 11 mM d-glucose was the designated minimal medium. Minimal medium was supplemented with 1 mM l-isoleucine or 1 mM glycine, as indicated. All chemicals were purchased from the Sigma-Aldrich Chemical Company (St. Louis, MO).

### Generation of cell pellets and spent media.

Ten biologically independent wild-type and *ridA* mutant cultures were grown overnight in NB medium with shaking at 37°C and used to inoculate (1% inoculum) 250 ml nonbaffled flasks holding 125 ml of medium. Each culture inoculated one each of the three medium types (minimal medium, minimal medium with 1 mM l-isoleucine, and minimal medium with 1 mM glycine), for a total of 60 flasks. Flasks were randomly arranged in an Innova 44 incubator, and cultures were allowed to grow for 16 h at 37°C, with shaking at 180 rpm. Cultures were chilled 5 min on ice and then harvested by centrifugation at 7,000 × *g* for 10 min at 4°C. The supernatant was decanted, with 10 ml transferred to sterile 15-ml conical tubes and flash-frozen using liquid nitrogen for downstream analysis of external metabolites. Cell pellets were transferred to sterile 15-ml conical tubes after resuspension in 10 ml double-distilled water, prior to a second pelleting at 7,000 × *g* for 10 min at 4°C. The supernatant was decanted, and pellets were flash-frozen using liquid nitrogen and stored at –80°C.

### Preparation of growth medium samples.

Spent medium from each bacterial culture was lyophilized (VirTis Benchtop K) for 48 h. Once dry, each lyophilized sample was reconstituted in 150 μl of deuterated (D_2_O, D, 99.9%; Cambridge Isotope Laboratories) 100 mM sodium phosphate buffer (mono- and dibasic; Fisher BioReagents), pH 7.0, containing 0.33 mM DSS (sodium 2,2-dimethyl-2-silapentane-5-sulfonate, D6, 98%; Cambridge Isotope Laboratories) as an internal standard. Each sample was centrifuged at 20,000 × *g* for 30 min, and 50 μl of supernatant was transferred by a Bruker SamplePro liquid handler into 1.7-mm SampleJet NMR tubes (Bruker Biospin).

### Metabolite extraction from bacterial pellets.

Each frozen bacterial pellet was thawed on ice and 1 ml of ice-cold 80:20 methanol-water, together with approximately 200 ml of 0.7-mm silica beads (BioSpec Products). Homogenization was carried out using a FastPrep 96 (MPBIO). The samples and extraction blanks went through three cycles of homogenization at 1,800 rpm for 300 s each. At the end of each cycle, samples and controls were centrifuged at 20,000 × *g* for 30 min. Each supernatant was transferred to a new tube and 1 ml of ice-cold methanol-water added to the original tubes before each new cycle. The combined supernatants from each cycle were pooled and concentrated overnight using a CentriVap benchtop vacuum concentrator (Labconco) down to 0.1 ml. The samples then were diluted with 0.5 ml of methanol-water, transferred into 0.6-ml centrifuge tubes, and concentrated to dryness. The extracts were reconstituted in 150 μl of deuterated 100 mM sodium phosphate buffer containing 0.33 mM the internal standard DSS at pH 7.0 and vortex mixed for 5 min. Each sample was centrifuged at 20,000 × *g* for 30 min and transferred by a Bruker SamplePro liquid handler into 1.7-mm SampleJet NMR tubes. Extraction blanks were prepared by following the same procedure, except the biological material was replaced with an equal volume of water. Solvent blanks consisted of the reconstituting NMR buffer (deuterated sodium phosphate buffer with DSS).

### Acquisition and processing of NMR data.

One-dimensional (1D) ^1^H NMR spectra for each sample and blanks were acquired using an optimized PROJECT (periodic refocusing of *J* evolution by coherence transfer) pulse sequence (42) on an Avance III HD 600 MHz Bruker NMR spectrometer equipped with a TCI cryoprobe and a Bruker SampleJet autosampler cooled to 5.6°C. During acquisition, 32,768 complex data points were collected using 64 scans with 16 additional dummy scans. The spectral width was 20 ppm. A Fourier transform (FT), a polynomial baseline correction of order 3, a 2-Hz line broadening, and phase correction were applied to each spectrum.

Two-dimensional ^1^H-^1^H total correlation spectroscopy (TOCSY; dipsi2esfbgpph), ^1^H-^13^C heteronuclear single quantum correlation (HSQC; hsqcedetgpsisp2.3), and ^1^H-^13^C HSQC-total correlation spectroscopy (HSQC-TOCSY; hsqcdietgpsisp.2) experiments were collected on pooled samples, composed of a small aliquot from each study sample, for metabolite identification. During acquisition, all three experiments were collected for 32 scans and an additional 16 dummy scans, with 512 and 1,024 data points recorded on the direct and indirect dimensions, respectively, and a spectral width of 200 ppm for ^13^C and 12 ppm for ^1^H. A 90-ms mixing time was used for both HSQC-TOCSY and TOCSY experiments. All spectral processing was carried out using NMRpipe (43). Spectrum referencing, baseline correction, and statistical analysis were carried out using in-house MATLAB (Mathworks R2019a) scripts.

### Compound identification/database matching.

All three two-dimensional experiments were used for spectral matching against the COLMARm database (44) using chemical shift cutoffs of 0.03 and 0.3 ppm for ^1^H and ^13^C, respectively. A total of 9 exogenous and 16 endogenous metabolites that could be integrated without overlapping features in their respective 1D ^1^H NMR spectra were identified. A confidence level ranging from 1 to 5 was assigned to each metabolite (see Tables S1 and S2 in the supplemental material), as described elsewhere (45). Briefly, this scale is defined as (i) putatively characterized compound, (ii) matched reported 1D spectra, (iii) matched reported HSQC spectra, (iv) matched reported HSQC and HSQC-TOCSY spectra, and (v) validation by spiking a synthetic standard into the sample.

### Statistical analysis.

The data were normalized using probabilistic quotient normalization (PQN) and range-scaled before multivariate statistical analysis (46, 47). The principal component analysis (PCA) scores were calculated using the NIPALS algorithm (48). The PLS-DA using the SIMPLS algorithm was conducted with a 5-fold cross-validation and 30 permutations (49). Goodness of prediction (*Q*^2^) for the PLS-DA model was obtained, and the model was used to identify features that differed between the wild type and *ridA* mutant for both endogenous and exogenous data sets (50). VIP scores from the PLS-DA data were determined using the “vip” function from PLS_Toolbox (Eigenvector Research) (30). This provided a list of VIP scores corresponding to every points-per-million data point contributing to the spectra. This list was trimmed to only contain VIP scores for every spectral peak in the data set, and known chemical IDs were matched to these peaks (Tables S3 and S4). The VIP scores provided in Fig. 3 represent the average VIP score from all of the nonoverlapped peaks belonging to the given metabolite. Univariate statistics were performed using PQN-normalized 1D ^1^H NMR data for metabolites whose features could be integrated without the presence of overlapping features. Student's *t* test with a Benjamini-Hochberg false discovery rate (FDR) correction (51) was used to determine metabolites that differed significantly (*q* value of <0.1) between wild-type and *ridA* mutant samples. All raw and processed data are available on the Metabolomics Workbench (www.metabolomicsworkbench.org), along with detailed experimental NMR and statistical analysis methods.

### Data availability.

All raw and processed data, along with detailed experimental NMR and statistical analysis methods, are available at the NIH Common Fund’s National Metabolomics Data Repository (NMDR) website, the Metabolomics Workbench (www.metabolomicsworkbench.org, under project identifier PR000889). The data can be accessed directly via its project doi, https://doi.org/10.21228/M8S39G. Metabolomics Workbench is supported by NIH grant U2C-DK119886. All in-house MATLAB scripts used are publicly available at https://github.com/artedison/Edison_Lab_Shared_Metabolomics_UGA.
