# Supplementary figures and images for: Proton Nuclear Magnetic Resonance Metabolomics Corroborates Serine Hydroxymethyltransferase as the Primary Target of 2-Aminoacrylate in a ridA Mutant of Salmonella enterica
Source: mSystems. 2020 Mar 10;5(2):e00843-19. doi: 10.1128/mSystems.00843-19 (PMC7065518; doi:10.1128/mSystems.00843-19)

**A**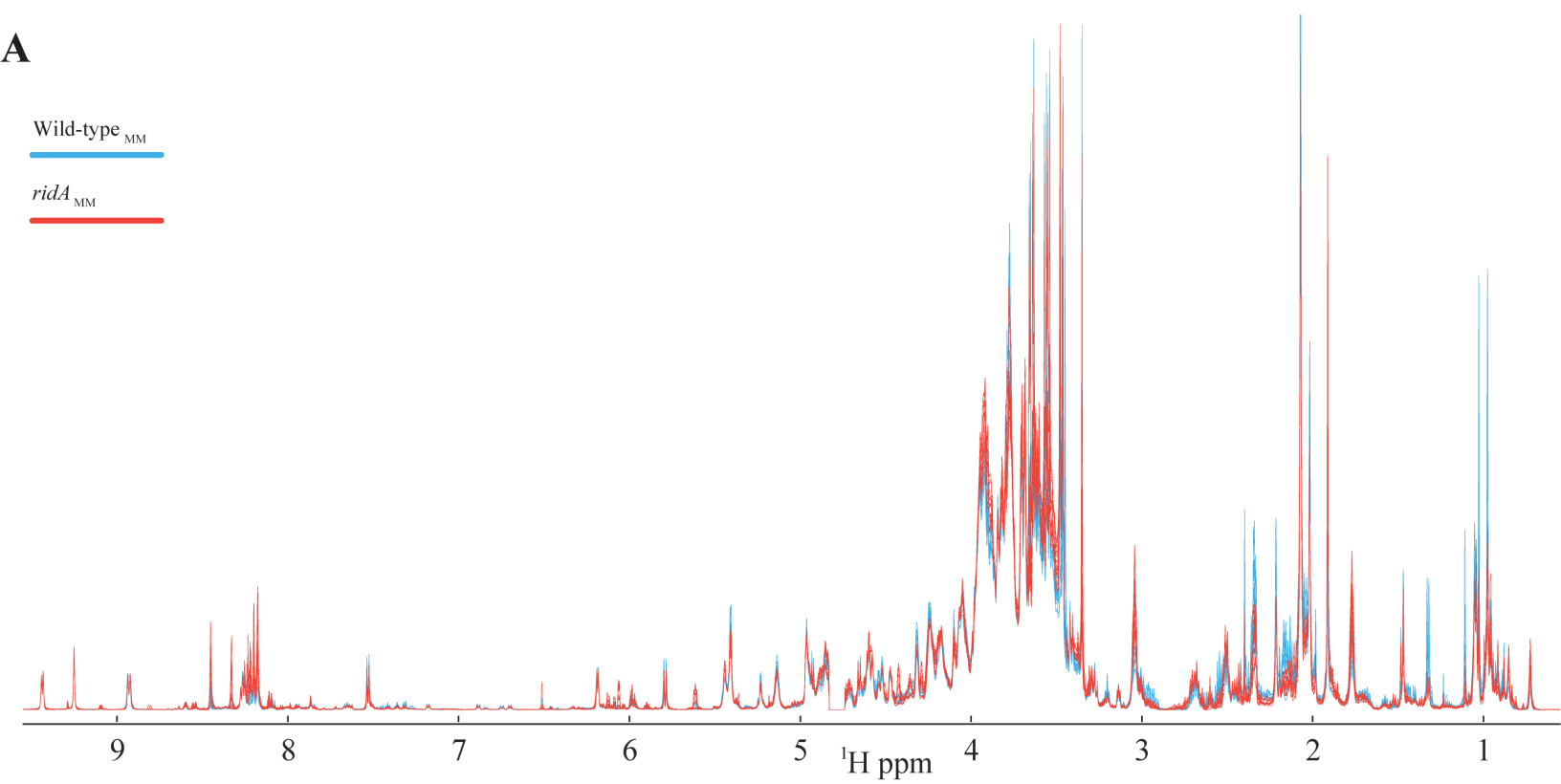**B**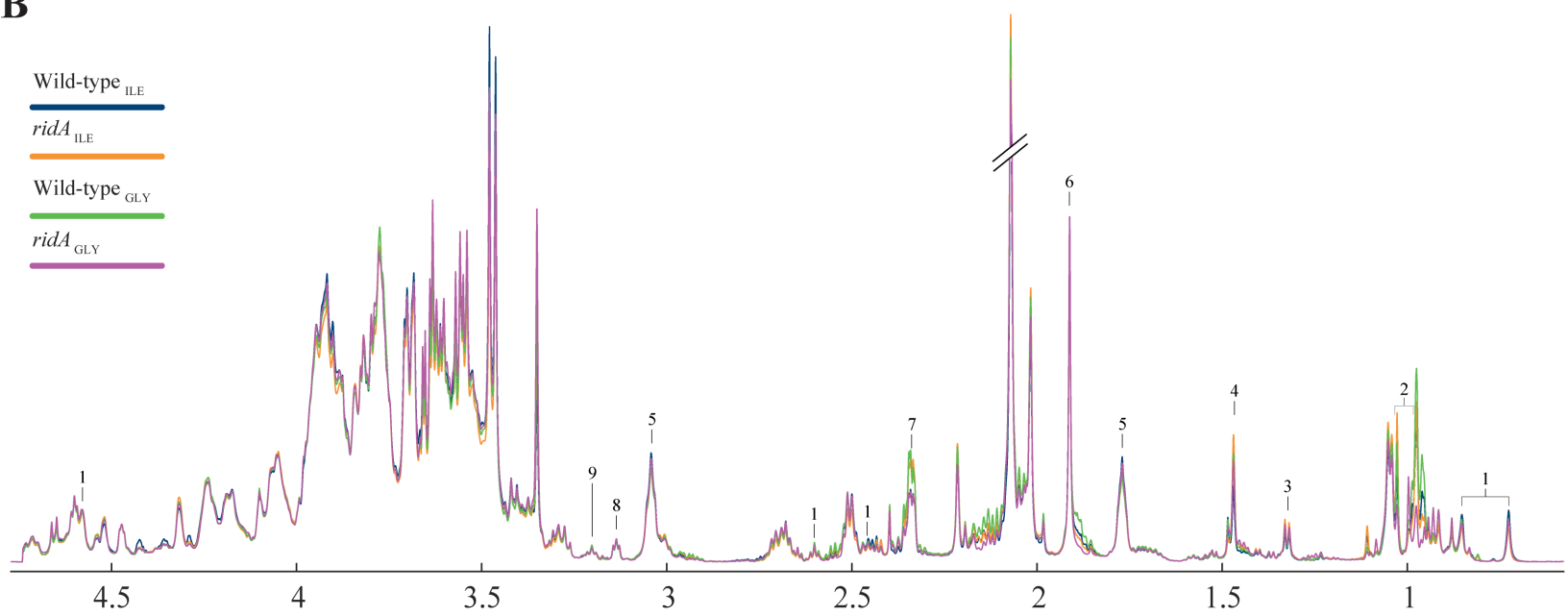**C**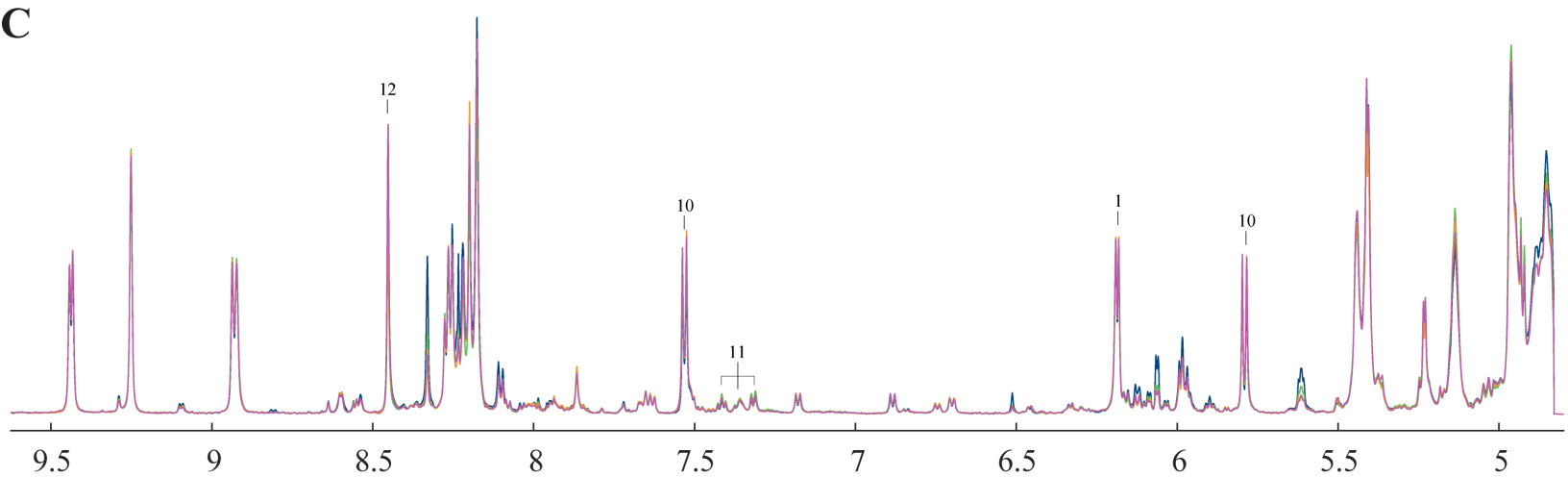

Supplement: FIG S1 [file mSystems.00843-19-sf001.pdf]

**A**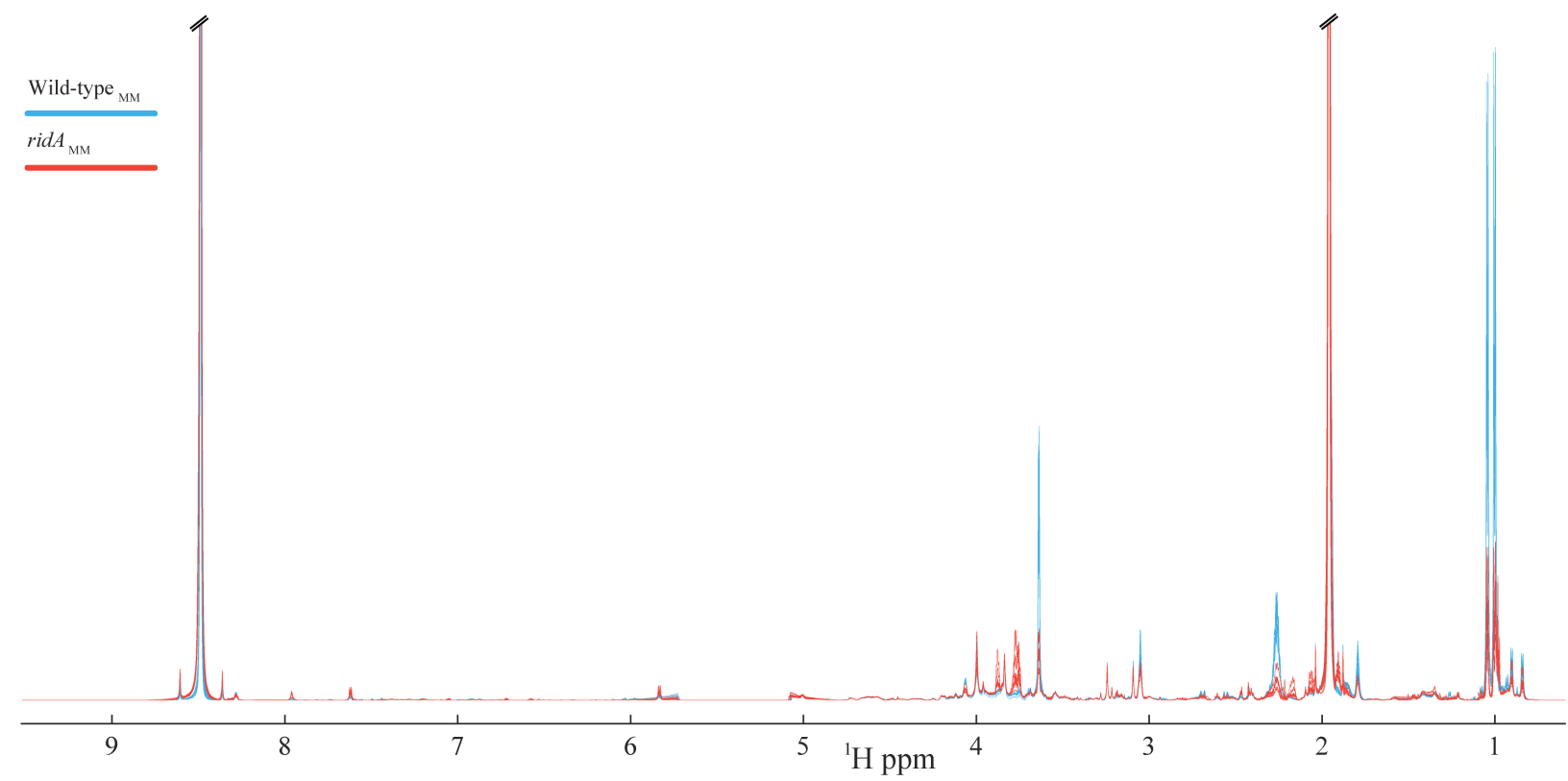**B**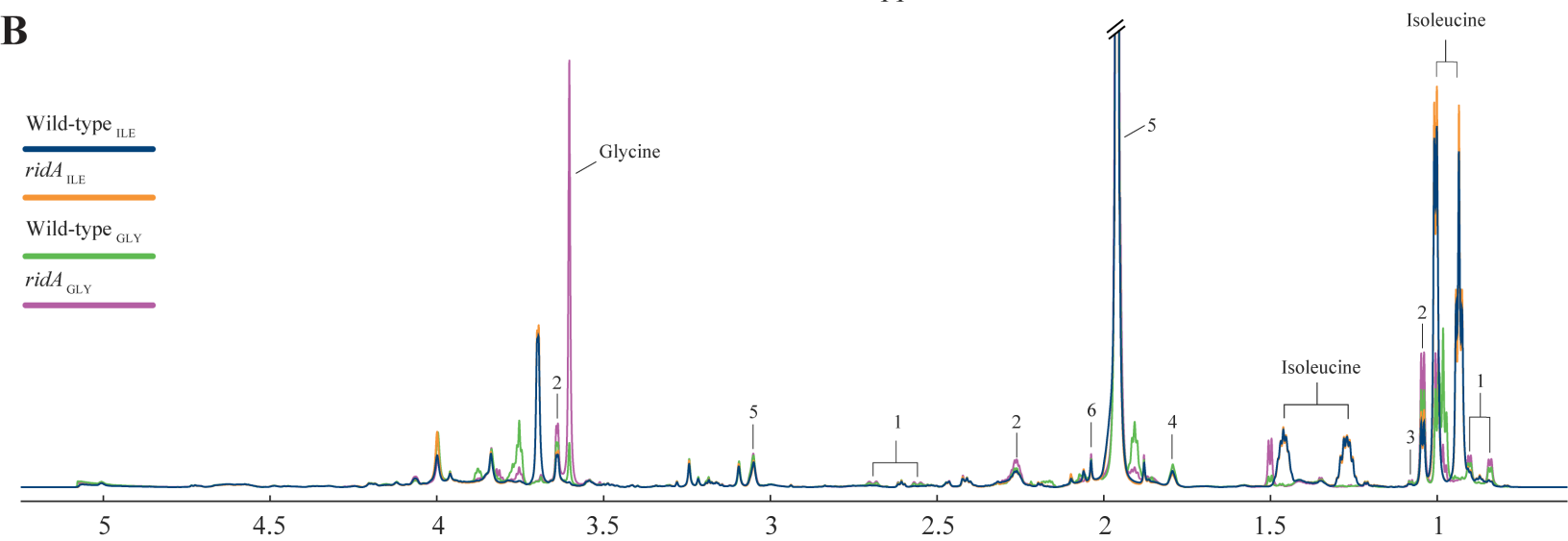**C**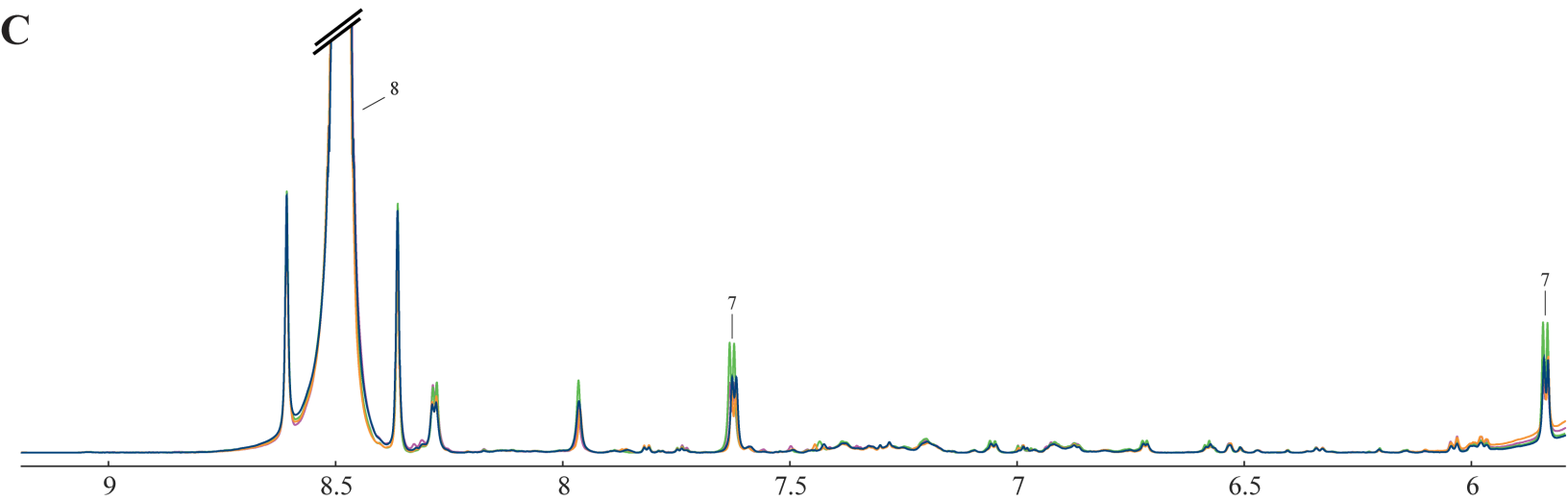

Supplement: FIG S2 [file mSystems.00843-19-sf002.pdf]

**A**

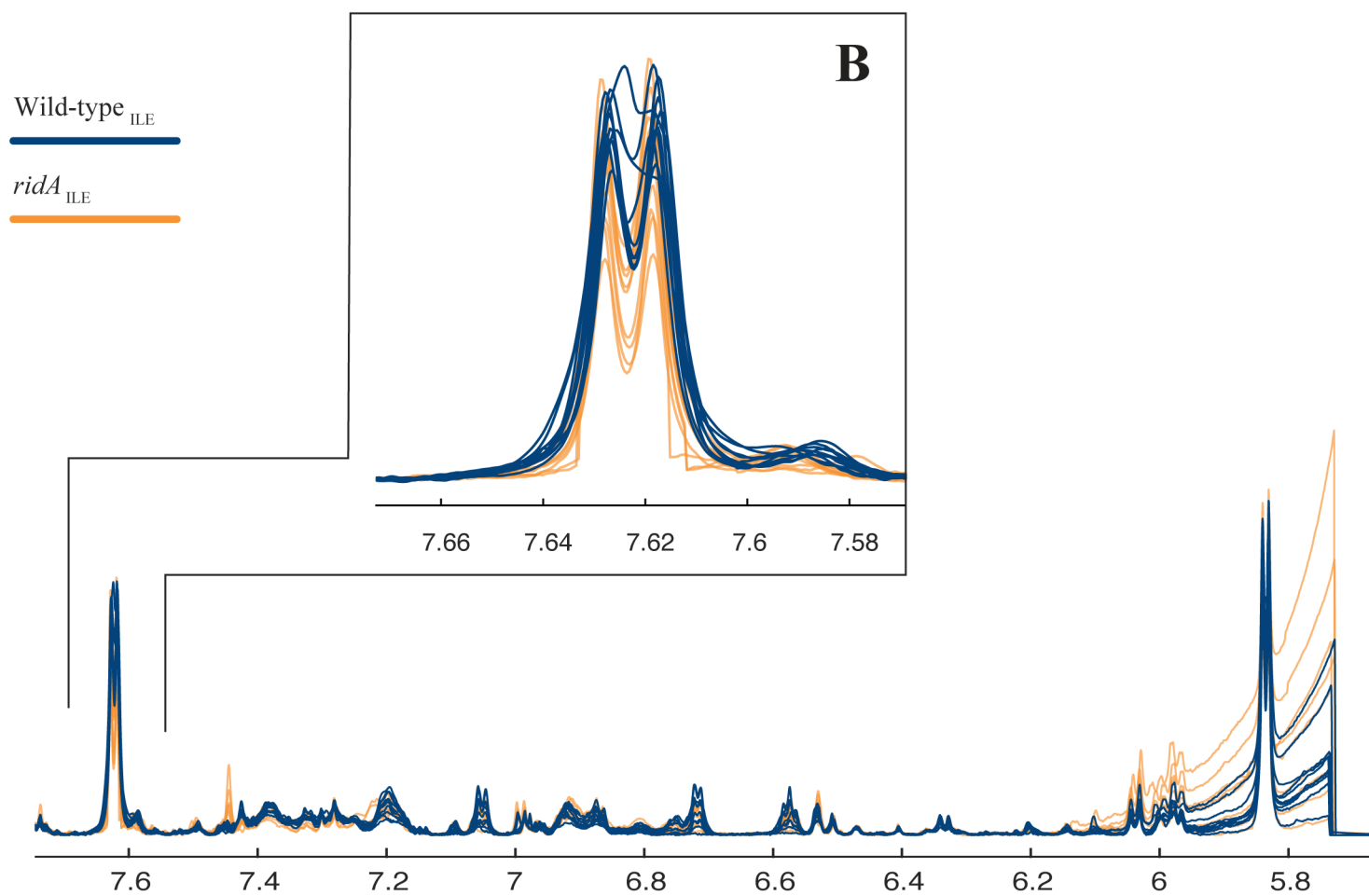

Supplement: FIG S3 [file mSystems.00843-19-sf003.pdf]
