# Supplementary material for: Proton Nuclear Magnetic Resonance Metabolomics Corroborates Serine Hydroxymethyltransferase as the Primary Target of 2-Aminoacrylate in a ridA Mutant of Salmonella enterica
Source: mSystems. 2020 Mar 10;5(2):e00843-19. doi: 10.1128/mSystems.00843-19 (PMC7065518; doi:10.1128/mSystems.00843-19)
Supplement: TABLE S1 [file mSystems.00843-19-st001.pdf]

Table S1. Endogenous Metabolites Identified by ^{1}H-NMR in Pellet Samples with Confidence Levels

Endogenous Metabolites

Metabolite	Assignment	^{1}H Chemical shift peaks (ppm) [Multiplicity]	Confidence Level
Acetate	CH_3	1.912 [s]	4
Alanine	CH_3	1.469, 1.484 [d]	4
Coenzyme A	CH_3	0.726 [s]	4
	CH_3	0.853 [s]	
	CH_2	2.445, 2.456, 2.470 [t]	
	CH_2	3.308 [s]	
	CH	6.182, 6.191 [d]	
Ethanolamine	CH_2NH_2	3.128, 3.136, 3.145 [t]	4
Formate	CH	8.453 [s]	4
Glutamate	αCH_2	2.017 [m]	4
	βCH_2	2.334-2.346 [m]	
	CH	3.774 [m]	
Glutamine		2.170 [s]	4
N-acetylputrescine	CH_2	1.565-1.602 [m]	4
	CH_2	1.667-1.695 [m]	
	CH_3	1.983 [s]	
	CH_2NH_2	2.992, 3.006, 3.019 [t]	
	CH_2	3.192, 3.203, 3.214 [t]	

Endogenous Metabolites

Metabolite	Assignment	^{1}H Chemical shift peaks (ppm) [Multiplicity]	Confidence Level
Nicotinate		8.596 [s]	4
		8.603 [s]	
		9.289 [s]	
Phenylalanine	CH	7.311, 7.323 [d]	4
	CH	7.349-7.374 [m]	
	CH	7.403-7.428 [m]	
Putrescine	CH_2	1.770 [m]	4
	CH_2NH_2	3.042 [t]	
Pyruvate	CH_3	2.359 [s]	4
Succinate	CH_2	2.398 [s]	4
Threonine	CH_3	1.319, 1.330 [d]	4
	CH	4.238-4.245 [m]	
Uracil	CH	5.784, 5.798 [d]	4
	CHNH	7.525, 7.537 [d]	
Valine	γCH_3	0.976 [d]	4
	γCH_3	1.028 [d]	
	αCH	3.653, 3.66 [d]	
	βCH	2.251-2.291 [m]	
