# Supplementary material for: Proton Nuclear Magnetic Resonance Metabolomics Corroborates Serine Hydroxymethyltransferase as the Primary Target of 2-Aminoacrylate in a ridA Mutant of Salmonella enterica
Source: mSystems. 2020 Mar 10;5(2):e00843-19. doi: 10.1128/mSystems.00843-19 (PMC7065518; doi:10.1128/mSystems.00843-19)
Supplement: TABLE S2 [file mSystems.00843-19-st002.pdf]

Table S2. Exogenous Metabolites Identified by ^{1}H-NMR in Medium Samples with Confidence Levels

Exogenous Metabolites				***Exogenous Metabolites***			
Metabolite	Assignment	^{1}H Chemical shift peaks (ppm) [Multiplicity]	Confidence Level	Metabolite	Assignment	^{1}H Chemical shift peaks (ppm) [Multiplicity]	Confidence Level
Lactic acid	CH_3	1.259, 1.266 [d]	4	2-isopropylmalic acid	CH_2	2.547, 2.567, 2.681, 2.700 [d of d]	4
	CH	4.062 [q]			CH_3	0.838, 0.845 [d]	
Valine	αCH	3.636, 3.641 [d]	4		CH_3	0.899, 0.907 [d]	
	βCH	2.253-2.302 [m]		Putrescine	CH_2	1.793 [m]	4
	γCH_3	0.997, 1.006 [d]			CH_2NH_2	3.050 [t]	
	γCH_3	1.039, 1.048 [d]		2-aminobutyric acid	CH_3	0.974, 0.984 [t]	4
Isoleucine	αCH	3.63, 3.64 [d]	4		CH_2	1.90, 1.91, 1.92 [m]	
	CH_2	1.45-1.48 [m]			CH	3.75, 3.75, 3.76 [t]	
	CH_2	1.25-1.30 [m]		Acetate	CH_3	1.96 [s]	4
	CH_3	.926, 0.935, 0.944 [t]		acetyl-phosphate	CH_3	2.095 [s]	4
	CH_3	1.00, 1.01 [d]		Uracil	CH	5.830, 5.839 [d]	4
Formate	CH	8.488 [s]	4		CHNH	7.617, 7.625 [d]	
