# Supplementary material for: Proton Nuclear Magnetic Resonance Metabolomics Corroborates Serine Hydroxymethyltransferase as the Primary Target of 2-Aminoacrylate in a ridA Mutant of Salmonella enterica
Source: mSystems. 2020 Mar 10;5(2):e00843-19. doi: 10.1128/mSystems.00843-19 (PMC7065518; doi:10.1128/mSystems.00843-19)
Supplement: TABLE S3 [file mSystems.00843-19-st003.pdf]

Table S3. VIP Scores for Endogenous PLS-DA Plot Component 1

Peak ppm	VIP Score	Peak Identity		Peak ppm	VIP Score	Peak Identity
1.409	2.92			1.565	1.84	N-acetyl putrescine
1.990	2.76			3.192	1.83	N-acetyl putrescine
1.398	2.67			5.010	1.81	
2.500	2.55			7.458	1.81	
1.319	2.52	Threonine		2.489	1.81	
3.462	2.51			4.238	1.80	Threonine
1.330	2.51	Threonine		3.392	1.80	
5.441	2.51			7.720	1.78	
1.516	2.48			8.176	1.77	
2.679	2.43			3.774	1.76	Glutamate
3.479	2.36			5.032	1.75	
2.215	2.33			8.453	1.74	Formate
2.690	2.31			2.303	1.74	
2.667	2.31			3.708	1.73	
4.245	2.28	Threonine		7.428	1.73	Phenylalanine
3.902	2.27			5.017	1.71	
2.512	2.27			1.999	1.71	
2.251	2.27	Valine		3.948	1.71	
5.310	2.24			7.846	1.70	
1.430	2.24			7.415	1.69	Phenylalanine
3.918	2.23			1.288	1.68	
1.028	2.21	Valine		1.591	1.68	N-acetyl putrescine
1.527	2.19			3.622	1.66	
4.539	2.17			3.883	1.66	
3.145	2.13	Ethanolamine		7.311	1.65	Phenylalanine
0.889	2.11			1.454	1.63	
2.266	2.07	Valine		7.444	1.63	
2.717	2.06			6.849	1.62	
3.386	2.05			1.199	1.62	
2.291	2.04	Valine		1.667	1.62	N-acetyl putrescine
1.442	2.03			2.194	1.62	
3.136	2.01	Ethanolamine		2.557	1.59	
2.131	2.01			1.961	1.59	
1.983	1.99	N-acetyl putrescine		7.323	1.58	Phenylalanine
3.875	1.98			4.995	1.57	
3.612	1.97			2.545	1.56	
5.048	1.96			2.170	1.55	Glutamine
1.577	1.95	N-acetyl putrescine		3.006	1.55	N-acetyl putrescine
3.214	1.95	N-acetyl putrescine		6.877	1.54	
2.706	1.95			0.726	1.54	Coenzyme A
1.188	1.93			4.714	1.54	
3.602	1.92			1.680	1.54	N-acetyl putrescine
2.144	1.92			8.253	1.51	
0.976	1.91	Valine		7.895	1.50	
3.203	1.91	N-acetyl putrescine		6.153	1.48	
3.498	1.90			8.421	1.48	
5.319	1.88			1.469	1.48	Alanine
0.928	1.86			1.042	1.47	
2.622	1.85			8.638	1.47	
4.318	1.84			7.911	1.45	

Table S3 *continued*. VIP Scores for Endogenous PLS-DA Plot Component 1

Peak ppm	VIP Score	Peak Identity		Peak ppm	VIP Score	Peak Identity
2.747	1.44			2.316	1.20	
8.076	1.44			0.767	1.20	
1.052	1.44			2.789	1.20	
2.570	1.43			5.235	1.20	
2.964	1.43			2.342	1.18	Glutamate
3.733	1.39			2.346	1.18	Glutamate
7.403	1.39	Phenylalanine		8.088	1.18	
8.234	1.39			5.136	1.18	
2.757	1.39			0.880	1.18	
4.868	1.38			6.066	1.17	
1.770	1.38	Putrescine		4.922	1.17	
3.525	1.36			2.120	1.16	
7.666	1.36			8.043	1.16	
3.683	1.36			4.932	1.15	
2.992	1.36	N-acetyl putrescine		3.232	1.14	
3.375	1.36			4.209	1.14	
				1.695	1.13	N-acetyl putrescine
1.484	1.35	Alanine		2.390	1.13	
6.326	1.35			1.173	1.13	
8.109	1.35			8.031	1.13	
2.930	1.35			1.137	1.11	
6.836	1.34			3.261	1.11	
3.042	1.33	Putrescine		8.381	1.09	
7.374	1.33	Phenylalanine		1.148	1.09	
7.359	1.33	Phenylalanine		2.334	1.08	Glutamate
3.702	1.32			8.364	1.06	
2.071	1.32			2.918	1.06	
8.096	1.31			3.174	1.05	
2.048	1.30			1.855	1.05	
2.940	1.28			3.308	1.04	Coenzyme A
1.602	1.27	N-acetyl putrescine		7.673	1.04	
3.128	1.26	Ethanolamine		2.360	1.04	Pyruvate
2.955	1.26			8.937	1.03	
6.337	1.25			0.810	1.03	
4.478	1.25			3.228	1.03	
2.456	1.25	Coenzyme A		5.296	1.03	
2.157	1.24			2.636	1.03	
8.135	1.24			4.578	1.02	
1.246	1.24			4.424	1.01	
3.594	1.24			6.891	1.00	
2.017	1.24	Glutamate		7.651	1.00	
7.958	1.23			5.984	1.00	
8.330	1.22			7.185	1.00	
4.065	1.22			4.394	0.99	
6.059	1.22			7.172	0.99	
2.798	1.22			0.853	0.98	Coenzyme A
8.221	1.22			5.411	0.98	
3.980	1.22			4.608	0.98	
2.035	1.21			9.433	0.98	
5.229	1.21			2.979	0.97	

Table S3 *continued*. VIP Scores for Endogenous PLS-DA Plot Component 1

Peak ppm	VIP Score	Peak Identity		Peak ppm	VIP Score	Peak Identity
6.191	0.97	Coenzyme A		2.375	0.74	
4.883	0.96			3.277	0.73	
4.473	0.96			1.268	0.73	
4.185	0.96			5.899	0.72	
1.122	0.96			6.182	0.72	Coenzyme A
5.068	0.95			5.247	0.71	
8.923	0.95			1.912	0.71	Acetate
1.946	0.95			1.545	0.70	
3.450	0.94			6.693	0.70	
2.533	0.94			2.108	0.70	
3.350	0.94			4.594	0.70	
2.906	0.93			4.414	0.69	
5.784	0.93	Uracil		3.106	0.69	
1.281	0.92			0.916	0.69	
8.603	0.92	Nicotinate		4.683	0.68	
3.019	0.92	N-acetyl putrescine		6.287	0.67	
5.268	0.91			1.887	0.67	
1.374	0.91			4.292	0.66	
7.502	0.91			4.288	0.66	
5.969	0.90			2.810	0.66	
1.554	0.90			4.174	0.64	
4.600	0.90			3.827	0.63	
2.781	0.90			6.739	0.62	
1.161	0.90			8.596	0.62	Nicotinate
5.494	0.89			1.881	0.62	
6.753	0.89			9.442	0.61	
7.537	0.88	Uracil		4.131	0.60	
5.798	0.87	Uracil		1.624	0.60	
2.445	0.87	Coenzyme A		7.986	0.59	
8.199	0.87			4.351	0.59	
5.406	0.87			3.244	0.59	
8.265	0.87			7.936	0.58	
2.657	0.86			1.361	0.58	
1.108	0.86			5.607	0.58	
6.707	0.85			4.342	0.57	
1.216	0.84			0.956	0.57	
8.142	0.82			2.398	0.57	Succinate
6.167	0.81			2.601	0.57	
4.730	0.79			0.944	0.56	
1.892	0.79			3.292	0.56	
2.770	0.78			3.588	0.55	
5.911	0.78			8.404	0.55	
7.525	0.77	Uracil		7.489	0.55	
1.803	0.77			2.093	0.55	
4.380	0.76			7.998	0.54	
1.234	0.76			4.359	0.54	
4.050	0.76			5.623	0.54	
0.832	0.76			5.616	0.53	
9.251	0.74			7.624	0.53	
7.475	0.74			5.614	0.52	

Table S3 *continued*. VIP Scores for Endogenous PLS-DA Plot Component 1

Peak ppm	VIP Score	Peak Identity		Peak ppm	VIP Score	Peak Identity
3.317	0.52			7.950	0.28	
4.407	0.52			0.959	0.28	
5.851	0.51			4.661	0.28	
6.140	0.50			4.100	0.27	
5.504	0.49			3.571	0.27	
2.522	0.48			5.960	0.27	
5.888	0.48			3.559	0.26	
3.436	0.48			3.993	0.25	
1.707	0.48			6.083	0.24	
4.962	0.47			2.590	0.24	
5.363	0.47			3.162	0.22	
6.103	0.45			6.029	0.21	
8.559	0.45			2.422	0.19	
3.405	0.45			8.538	0.19	
1.636	0.45			8.276	0.18	
6.455	0.44			1.725	0.17	
2.470	0.44	Coenzyme A		6.296	0.15	
3.819	0.43			6.038	0.15	
1.501	0.43			6.302	0.14	
0.996	0.43			3.094	0.14	
8.119	0.42			8.551	0.14	
3.659	0.42	Valine		3.842	0.14	
4.853	0.42			5.944	0.13	
5.376	0.41			9.100	0.13	
7.866	0.41			3.540	0.13	
5.169	0.41			3.798	0.13	
2.612	0.40			3.550	0.13	
8.013	0.39			4.466	0.13	
5.089	0.38			6.130	0.13	
1.823	0.38			5.501	0.12	
1.740	0.38			7.832	0.12	
5.842	0.37			3.361	0.12	
3.653	0.36	Valine		9.090	0.08	
1.257	0.35			2.433	0.08	
4.739	0.35			1.084	0.07	
7.788	0.34			3.667	0.06	
4.520	0.34			9.289	0.06	Nicotinate
1.869	0.31			3.632	0.06	
4.839	0.31			3.420	0.05	
6.092	0.31			3.810	0.05	
5.183	0.31			5.873	0.04	
6.512	0.30			6.463	0.04	
6.275	0.29			2.646	0.04	
3.640	0.29			6.118	0.03	
7.639	0.29			4.648	0.02	
5.994	0.28			1.715	0.01	
3.790	0.28					
