# Supplementary material for: Proton Nuclear Magnetic Resonance Metabolomics Corroborates Serine Hydroxymethyltransferase as the Primary Target of 2-Aminoacrylate in a ridA Mutant of Salmonella enterica
Source: mSystems. 2020 Mar 10;5(2):e00843-19. doi: 10.1128/mSystems.00843-19 (PMC7065518; doi:10.1128/mSystems.00843-19)
Supplement: TABLE S4 [file mSystems.00843-19-st004.pdf]

Table S4. VIP Scores for Exogenous PLS-DA Plot Component 1

Peak ppm	VIP Score	Peak Identity		Peak ppm	VIP Score	Peak Identity
1.259	2.66	Lactate		0.838	1.75	2-isopropylmalate
7.000	2.64			6.988	1.74	
1.266	2.55	Lactate		7.351	1.74	
1.116	2.46			1.915	1.73	2-aminobutyrate
1.124	2.46			6.876	1.71	
1.108	2.43			3.050	1.71	Putrescine
0.963	2.39			0.845	1.71	2-isopropylmalate
7.494	2.38			3.350	1.69	
7.960	2.36			1.906	1.65	2-aminobutyrate
3.641	2.30	Valine		2.320	1.65	
6.938	2.28			7.424	1.65	
2.283	2.27	Valine		0.899	1.64	2-isopropylmalate
1.039	2.26	Valine		3.120	1.63	
1.074	2.26			3.341	1.63	
2.261	2.26	Valine		2.681	1.62	2-isopropylmalate
1.048	2.25	Valine		7.556	1.60	
5.967	2.25			6.868	1.60	
1.006	2.25	Valine		3.144	1.59	
2.275	2.24	Valine		2.700	1.59	2-isopropylmalate
0.997	2.22	Valine		1.793	1.58	Putrescine
0.925	2.21			2.472	1.57	
3.636	2.21	Valine		2.517	1.56	
2.253	2.19	Valine		0.907	1.56	2-isopropylmalate
2.917	2.18			6.201	1.56	
2.267	2.17	Valine		1.409	1.53	
2.922	2.14			4.155	1.53	
7.726	2.12			2.998	1.53	
5.977	2.09			2.095	1.52	Acetyl-phosphate
2.302	2.07	Valine		1.856	1.51	
1.084	2.04			3.441	1.51	
6.031	2.01			7.301	1.50	
0.955	2.00			3.747	1.50	2-aminobutyrate
6.044	1.99			4.574	1.50	
2.464	1.95			0.787	1.49	
8.362	1.94			3.753	1.49	2-aminobutyrate
2.904	1.93			2.650	1.48	
8.607	1.93			1.415	1.48	
8.488	1.92	Formate		0.936	1.47	
7.625	1.91	Uracil		2.435	1.47	
7.736	1.91			8.629	1.47	
4.062	1.90	Lactate		1.870	1.46	
5.830	1.86	Uracil		3.193	1.45	
7.820	1.85			2.567	1.44	2-isopropylmalate
0.871	1.84			4.197	1.43	
5.839	1.83	Uracil		4.148	1.43	
7.809	1.83			2.547	1.43	2-isopropylmalate
7.617	1.82	Uracil		0.795	1.41	
6.926	1.82			0.974	1.39	2-aminobutyrate
7.440	1.79			4.597	1.37	
3.364	1.78			2.606	1.36	

Table S4 *continued*. VIP Scores for Exogenous PLS-DA Plot Component 1

Peak ppm	VIP Score	Peak Identity		Peak ppm	VIP Score	Peak Identity
3.185	1.33			1.180	0.96	
2.133	1.32			2.597	0.96	
2.527	1.32			2.065	0.95	
7.747	1.32			3.876	0.94	
3.760	1.31	2-aminobutyrate		2.225	0.91	
8.287	1.30			5.993	0.91	
8.279	1.30			4.324	0.91	
1.400	1.27			3.919	0.90	
2.444	1.26			2.056	0.90	
3.696	1.25			8.383	0.89	
0.984	1.25	2-aminobutyrate		1.388	0.88	
6.508	1.23			5.727	0.88	
2.616	1.22			7.281	0.87	
4.250	1.21			3.901	0.87	
4.275	1.18			0.944	0.86	
3.429	1.18			3.510	0.86	
8.678	1.15			1.731	0.85	
3.687	1.14			1.863	0.84	
2.730	1.14			2.171	0.84	
4.143	1.13			4.092	0.84	
4.590	1.12			5.740	0.82	
7.344	1.11			3.016	0.82	
8.304	1.10			5.942	0.81	
6.585	1.10			2.012	0.81	
4.513	1.09			2.836	0.81	
2.421	1.08			3.542	0.80	
2.313	1.08			1.722	0.79	
4.370	1.07			3.414	0.79	
4.345	1.07			2.400	0.78	
6.722	1.06			3.869	0.77	
3.784	1.06			4.861	0.77	
7.057	1.05			1.379	0.77	
6.566	1.05			2.183	0.76	
1.623	1.05			4.204	0.76	
7.046	1.05			5.939	0.76	
2.501	1.05			6.893	0.75	
3.700	1.05			2.345	0.75	
1.367	1.04			2.722	0.75	
4.193	1.04			3.726	0.73	
6.575	1.03			4.485	0.73	
3.769	1.03			4.622	0.73	
6.714	1.02			7.590	0.70	
2.163	1.02			5.947	0.70	
3.777	1.02			3.930	0.70	
3.176	1.01			5.735	0.69	
3.883	1.00			3.331	0.69	
1.217	0.99			8.327	0.68	
6.916	0.98			5.067	0.67	
1.209	0.98			4.456	0.66	
3.798	0.97			4.580	0.64	

Table S4 *continued*. VIP Scores for Exogenous PLS-DA Plot Component 1

Peak ppm	VIP Score	Peak Identity	Peak ppm	VIP Score	Peak Identity
6.001	0.62		1.234	0.30	
7.598	0.59		5.082	0.30	
3.155	0.56		2.371	0.30	
4.358	0.56		3.004	0.27	
3.589	0.54		4.887	0.27	
5.069	0.53		3.216	0.27	
1.533	0.51		1.188	0.25	
3.672	0.51		3.475	0.25	
3.579	0.51		3.243	0.25	
1.578	0.50		4.878	0.24	
3.499	0.49		7.249	0.23	
1.575	0.48		2.073	0.22	
4.040	0.47		4.185	0.22	
1.960	0.46	Acetate	3.960	0.21	
2.207	0.46		2.751	0.18	
4.565	0.46		4.916	0.17	
1.245	0.45		4.381	0.16	
2.195	0.45		2.938	0.16	
4.445	0.44		1.138	0.16	
7.377	0.43		3.837	0.15	
1.445	0.42		6.860	0.14	
3.944	0.42		6.341	0.14	
3.459	0.42		3.280	0.13	
4.715	0.41		7.416	0.13	
2.821	0.41		2.352	0.11	
3.093	0.41		3.025	0.11	
1.353	0.41		1.281	0.10	
2.789	0.39		1.465	0.09	
2.490	0.39		2.410	0.09	
2.805	0.39		6.328	0.08	
2.217	0.39		4.724	0.08	
3.168	0.38		1.692	0.07	
3.161	0.38		5.006	0.07	
1.552	0.38		0.000	0.06	
1.879	0.38		1.608	0.05	
7.384	0.37		1.290	0.05	
7.290	0.37		1.095	0.05	
4.422	0.36		4.735	0.05	
1.500	0.35		7.324	0.04	
3.487	0.34		1.489	0.04	
3.997	0.34		3.398	0.04	
7.203	0.34		3.302	0.03	
1.543	0.34		1.474	0.03	
6.531	0.34		3.308	0.03	
7.380	0.31		4.119	0.03	
3.601	0.30		3.711	0.02	
2.038	0.30		1.685	0.00	
