# Supplementary material for: Proton Nuclear Magnetic Resonance Metabolomics Corroborates Serine Hydroxymethyltransferase as the Primary Target of 2-Aminoacrylate in a ridA Mutant of Salmonella enterica
Source: mSystems. 2020 Mar 10;5(2):e00843-19. doi: 10.1128/mSystems.00843-19 (PMC7065518; doi:10.1128/mSystems.00843-19)
Supplement: TABLE S5 [file mSystems.00843-19-st005.pdf]

Table S5 -Endogenous metabolites integration values and descriptive statistics

Minimal

	Coenzyme A	Valine	Threonine	Alanine	Putrescine	Acetate	Glutamine	Glutamate	Pyruvate	Succinate	Ethanolamine	N-acetylputrescine	Uracil	Phenylalanine	Formate	Nicotinate
Peak_ppm	0.726	1.028	1.331	1.470	1.770	1.912	2.170	2.342	2.360	2.399	3.137	3.203	5.798	7.311	8.453	8.596
WT_mean	0.305	0.149	0.155	0.214	0.921	1.026	0.079	0.507	0.028	0.083	0.144	0.072	0.169	0.021	0.216	0.028
WT_Stdev	0.057	0.057	0.021	0.082	0.270	0.331	0.027	0.167	0.008	0.061	0.014	0.018	0.046	0.006	0.085	0.009
Mut_mean	0.189	0.518	0.486	0.304	0.593	0.691	0.190	0.747	0.037	0.121	0.110	0.138	0.216	0.039	0.057	0.034
Mut_Stdev	0.037	0.130	0.155	0.051	0.201	0.243	0.134	0.205	0.014	0.114	0.013	0.042	0.077	0.010	0.034	0.008
Fold_Change	0.619	3.466	3.127	1.418	0.644	0.674	2.402	1.474	1.310	1.444	0.763	1.918	1.280	1.830	0.263	1.228
p_value	3.67E-05	1.72E-07	2.82E-06	9.00E-03	6.34E-03	1.88E-02	1.94E-02	1.00E-02	1.01E-01	3.76E-01	1.92E-05	2.56E-04	1.12E-01	1.19E-04	3.43E-05	1.12E-01
FDR_value	1.17E-04	2.75E-06	2.25E-05	1.60E-02	1.27E-02	2.59E-02	2.59E-02	1.60E-02	1.20E-01	3.76E-01	1.02E-04	5.84E-04	1.20E-01	3.18E-04	1.17E-04	1.20E-01

Minimal_Gly

	Coenzyme A	Valine	Threonine	Alanine	Putrescine	Acetate	Glutamine	Glutamate	Pyruvate	Succinate	Ethanolamine	N-acetylputrescine	Uracil	Phenylalanine	Formate	Nicotinate
Peak_ppm	0.726	1.028	1.331	1.470	1.770	1.912	2.171	2.342	2.360	2.398	3.137	3.203	5.798	7.311	8.453	8.596
WT_mean	0.249	0.202	0.185	0.317	0.773	0.910	0.100	0.722	0.046	0.077	0.137	0.087	0.194	0.026	0.184	0.028
WT_Stdev	0.057	0.066	0.054	0.111	0.187	0.251	0.067	0.138	0.013	0.064	0.024	0.019	0.046	0.006	0.100	0.009
Mut_mean	0.190	0.259	0.229	0.418	0.784	1.018	0.116	0.736	0.037	0.089	0.142	0.083	0.189	0.033	0.156	0.029
Mut_Stdev	0.042	0.116	0.085	0.055	0.294	0.292	0.090	0.232	0.020	0.053	0.011	0.044	0.053	0.016	0.071	0.008
Fold_Change	0.761	1.282	1.240	1.318	1.014	1.118	1.164	1.019	0.807	1.157	1.037	0.958	0.975	1.292	0.846	1.011
p_value	1.56E-02	1.95E-01	1.80E-01	1.89E-02	9.23E-01	3.87E-01	6.52E-01	8.71E-01	2.51E-01	6.54E-01	5.50E-01	8.13E-01	8.28E-01	1.74E-01	4.77E-01	9.31E-01
FDR_value	1.51E-01	6.25E-01	6.25E-01	1.51E-01	9.31E-01	8.85E-01	9.31E-01	9.31E-01	6.69E-01	9.31E-01	9.31E-01	9.31E-01	9.31E-01	6.25E-01	9.31E-01	9.31E-01

Minimal_Ile

	Coenzyme A	Valine	Threonine	Alanine	Putrescine	Acetate	Glutamine	Glutamate	Pyruvate	Succinate	Ethanolamine	N-acetylputrescine	Uracil	Phenylalanine	Formate	Nicotinate
Peak_ppm	0.726	1.028	1.331	1.470	1.770	1.912	2.172	2.342	2.360	2.398	3.136	3.203	5.798	7.311	8.453	8.603
WT_mean	0.199	0.082	0.227	0.323	0.751	0.874	0.065	0.521	0.028	0.059	0.145	0.088	0.215	0.035	0.174	0.027
WT_Stdev	0.066	0.036	0.072	0.106	0.292	0.383	0.017	0.141	0.007	0.033	0.020	0.054	0.062	0.018	0.130	0.010
Mut_mean	0.248	0.081	0.211	0.286	0.901	1.146	0.068	0.483	0.029	0.079	0.152	0.065	0.202	0.028	0.248	0.025
Mut_Stdev	0.122	0.035	0.048	0.072	0.306	0.319	0.026	0.149	0.010	0.050	0.011	0.027	0.053	0.010	0.078	0.009
Fold_Change	1.242	0.988	0.931	0.885	1.199	1.311	1.046	0.926	1.013	1.344	1.048	0.742	0.940	0.786	1.430	0.940
p_value	2.84E-01	9.50E-01	5.78E-01	3.70E-01	2.77E-01	1.02E-01	7.68E-01	5.60E-01	9.26E-01	3.01E-01	3.47E-01	2.49E-01	6.21E-01	2.71E-01	1.37E-01	7.04E-01
FDR_value	6.57E-01	9.50E-01	8.28E-01	6.57E-01	6.57E-01	6.57E-01	8.77E-01	8.28E-01	9.50E-01	6.57E-01	6.57E-01	6.57E-01	8.28E-01	6.57E-01	6.57E-01	8.67E-01

Fold_change is expressed as (*ridA* /WT)
