# Supplementary material for: Proton Nuclear Magnetic Resonance Metabolomics Corroborates Serine Hydroxymethyltransferase as the Primary Target of 2-Aminoacrylate in a ridA Mutant of Salmonella enterica
Source: mSystems. 2020 Mar 10;5(2):e00843-19. doi: 10.1128/mSystems.00843-19 (PMC7065518; doi:10.1128/mSystems.00843-19)
Supplement: TABLE S6 [file mSystems.00843-19-st006.pdf]

Table S6 -Exogenous metabolites integration values and descriptive statistics

Minimal

	2-isopropylmalate	Valine	2-aminobutyrate	Acetate	Acetyl-phosphate	Putrescine	Lactate	Uracil	Formate
Peak_ppm	0.838	1.040	1.907	1.960	2.096	3.051	4.063	7.626	8.488
WT_mean	0.215	1.231	0.294	42.503	0.040	0.544	0.134	0.145	38.811
WT_Stdev	0.082	0.330	0.184	15.001	0.005	0.057	0.052	0.025	5.409
Mut_mean	0.510	6.118	N/A	42.479	0.054	0.789	0.274	0.046	14.080
Mut_Stdev	0.122	0.928	N/A	9.322	0.007	0.159	0.024	0.008	5.894
Fold_Change	2.368	4.971	N/A	0.999	1.356	1.449	2.044	0.319	0.363
p_value	5.55E-06	6.10E-12	Undetermined	9.97E-01	3.80E-05	2.38E-04	4.21E-07	7.52E-10	1.27E-08
FDR_value	9.99E-06	5.49E-11	Undetermined	9.97E-01	5.70E-05	2.68E-04	9.47E-07	3.39E-09	3.80E-08

Minimal_Gly

	2-isopropylmalate	Valine	2-aminobutyrate	Acetate	Acetyl-phosphate	Putrescine	Lactate	Uracil	Formate
Peak_ppm	0.839	1.040	1.908	1.961	2.097	3.051	4.064	7.633	8.489
WT_mean	0.270	1.364	0.985	32.242	0.041	0.504	0.072	0.139	34.911
WT_Stdev	0.095	0.505	0.394	17.838	0.008	0.083	0.016	0.020	5.383
Mut_mean	0.413	1.969	0.152	41.363	0.045	0.538	0.149	0.095	34.432
Mut_Stdev	0.209	0.447	0.135	13.936	0.004	0.160	0.024	0.026	7.504
Fold_Change	1.528	1.443	0.154	1.283	1.085	1.067	2.073	0.687	0.986
p_value	6.52E-02	1.10E-02	5.85E-06	2.19E-01	2.58E-01	5.62E-01	9.33E-08	6.21E-04	8.72E-01
FDR_value	1.17E-01	2.47E-02	2.63E-05	3.28E-01	3.31E-01	6.33E-01	8.40E-07	1.86E-03	8.72E-01

Minimal_Ile

	2-isopropylmalate	Valine	2-aminobutyrate	Acetate	Acetyl-phosphate	Putrescine	Lactate	Uracil	Formate	Isoleucine
Peak_ppm	0.846	1.040	1.921	1.962	2.099	3.051	4.065	7.619	8.489	1.271
WT_mean	0.048	0.824	N/A	30.452	0.044	0.387	0.112	0.103	33.419	1.858
WT_Stdev	0.039	0.307	N/A	15.542	0.004	0.043	0.034	0.014	3.208	0.193
Mut_mean	0.049	0.928	N/A	27.515	0.080	0.368	0.108	0.078	30.654	1.841
Mut_Stdev	0.036	0.357	N/A	15.970	0.066	0.088	0.021	0.018	11.684	0.165
Fold_Change	1.016	1.127	N/A	0.904	1.817	0.949	0.964	0.757	0.917	0.990
p_value	9.66E-01	5.07E-01	Undetermined	6.90E-01	1.20E-01	5.51E-01	7.56E-01	4.19E-03	5.02E-01	8.32E-01
FDR_value	9.66E-01	9.18E-01	Undetermined	9.25E-01	6.01E-01	9.18E-01	9.25E-01	4.19E-02	9.18E-01	9.25E-01

N/A- Peak for one or more samples was not detectable above baseline signal
Undetermined - Fold-change could not be calculated since one or both conditions did not show peak instensity above the baseline;
Fold_change is expressed as (*ridA* /WT)
